# BRAFV600E/pTERT double mutated papillary thyroid cancers exhibit immune gene suppression

**DOI:** 10.3389/fendo.2024.1440722

**Published:** 2024-12-09

**Authors:** Ana-Maria Sigarteu Chindris, Michael Rivera, Yaohua Ma, Asha Nair, Yi Liu, Xue Wang, Brian M. Necela, Jennifer M. Kachergus, John D. Casler, Christopher Brett, Ana M. Rivas Mejia, Victor J. Bernet, John A. Copland, Keith L. Knutson, E. Aubrey Thompson, Robert C. Smallridge

**Affiliations:** ^1^ Division of Endocrinology, Mayo Clinic, Jacksonville, FL, United States; ^2^ Division of Anatomic Pathology, Mayo Clinic, Rochester, MN, United States; ^3^ Department of Quantitative Health Sciences, Mayo Clinic, Jacksonville, FL, United States; ^4^ Department of Cancer Biology, Mayo Clinic, Jacksonville, FL, United States; ^5^ Department of Otorhinolaryngology/Audiology, Mayo Clinic, Jacksonville, FL, United States; ^6^ Department of Internal Medicine, Mayo Clinic, Jacksonville, FL, United States; ^7^ Department of Immunology, Mayo Clinic, Jacksonville, FL, United States

**Keywords:** papillary thyroid cancer (PTC), BRAF mutation V600 E, TERT promoter mutation (pTERT), immune genes, lymphocytic infiltration

## Abstract

**Introduction:**

BRAFV600E mutation (BRAF^mut^) is common in papillary thyroid cancer (PTC), and most patients have an excellent outcome. However, a TERT-promoter mutation (pTERT^mut^) in the presence of BRAF^mut^ (BRAF^mut^pTERT^mut^) has been demonstrated to confer a more aggressive behavior to PTC. Lymphocytic infiltration is often present in PTC. In this study, we sought to decipher the relationship between the BRAF and pTERT mutations and immune gene dysregulation in tumor samples from a cohort of 147 samples of PTC.

**Methods:**

The abundance of 770 immune gene transcripts was determined by multiprex capture/detection and digital counting of mRNA transcripts using the NanoString nCounter^®^ PanCancer Immune Profiling Panel.

**Results:**

We identified 40 immune transcripts differentially expressed in BRAF^mut^pTERT^mut^ *vs* BRAF^mut^pTERT wildtype (pTERT^wt^) (*P*<0.05). Transcripts induced by BRAF^mut^ alone were significantly repressed in BRAF^mut^pTERT^mut^ samples, such as genes expressed by lymphoid cells, antigen-presenting cells, and cytotoxic cells, including chemokines, cytokines, checkpoint control proteins, interferon downstream markers, TNF superfamily proteins and BMP markers. A validation analysis using 444 samples from The Cancer Genome Atlas (TCGA) PTC dataset yielded similar results. Deconvolution analysis confirmed differences in the immune cell populations such as increased presence of M2 macrophages in the BRAF^mut^pTERT^mut^ Mayo cohort and a lower abundance of M1 macrophages in the BRAF^mut^pTERT^mut^ TCGA cohort compared to BRAF^mut^pTERT^wt^. Most of the immune gene pathways were enriched in the BRAF^mut^pTERT^wt^ tumors in both Mayo and TCGA cohorts but not in BRAF^mut^pTERT^mut^. BRAF^mut^pTERT^wt^ had higher stromal lymphocytes infiltration as compared to BRAF^wt^pTERT^wt^ tumors, corroborating the transcriptomic findings.

**Discussion:**

To our knowledge this is the first report of a potential link between TERT and the immune microenvironment, offering an explanation for the aggressive nature of BRAF^mut^pTERT^mut^ PTC.

## 1 Introduction

Thyroid cancer has increased in incidence and mortality in the United States over the last few decades, with papillary thyroid cancer (PTC) as the most common histological subtype (1). While most PTCs are diagnosed early and highly curable, about 10% have an aggressive course, leading to multiple recurrences, distant metastatic disease, and eventually death. The molecular features responsible for tumor aggressiveness in PTC have not yet been fully deciphered.

The most common genetic alteration in PTC is BRAF V600E mutation (BRAF^mut^), which plays an important role in tumorigenesis (2). While most patients with PTC, including those with BRAF^mut^, have excellent outcomes, BRAF^mut^ in the presence of a C228T or C250T TERT-promoter mutation (pTERT^mut^) appears to confer a more aggressive phenotype to PTC than BRAF^mut^ alone (3). Interestingly, 12% to 18% of patients with PTC have BRAF^mut^pTERT^mut^, similar to the percentage of patients with PTC who experience poor outcomes (3, 4). To date, there is insufficient understanding of how the presence of BRAF^mut^pTERT^mut^ results in an aggressive PTC phenotype.

BRAF is a key regulator of growth factor/MAPK signaling, and one might expect that the constitutively active BRAF mutation might be a powerful oncogenic driver, such as RAS, RAF, ERBB2, and EGFR. However, the link between BRAF^mut^ and clinical aggressiveness of PTC has proved to be difficult to establish. In general, BRAFV600E does not appear to be an independent predictor of PTC-associated mortality (5). Despite that, it has been reported that BRAF^mut^ is associated with a more aggressive phenotype in patients older than 55 years of age (6, 7) and may be an independent risk factor for recurrence in this population (8).

The relationship between age, BRAF mutation status, and tumor aggressiveness is further complicated by the observation that pTERT^mut^ is associated with older age at diagnosis (9–11). Since earlier studies did not consider pTERT^mut^ a variable, it is possible that the relationship between BRAF^mut^ and age at diagnosis may reflect an age-associated increase in double mutant tumors, which express both BRAF^mut^ and pTERT^mut^. Such tumors have been shown to be larger, have a higher degree of extrathyroidal extension and are more aggressive than PTC tumors with wild type BRAF (BRAF^wt^) or BRAF^mut^ alone (3, 10).

The relationship between chronic lymphocytic thyroiditis and differentiated thyroid cancer has been the subject of ongoing debate. While up to 35% of PTC arise on a background of lymphocytic thyroiditis (BLT) (12), the effect of the immune microenvironment on the clinicopathological features of PTC is less clear, owing to the complexity of interactions between the tumor and different lymphocyte subsets (12–21). Nevertheless, the enormous impact of tumor immune microenvironment on cancer cell biology is now well established and targeted for therapeutic purposes in several malignancies, and to some extent, in advanced thyroid malignancies (22).

BRAF^mut^ is common in PTC with a reported prevalence of 48% in a cohort of 2,099 patients with PTC (7) and about 60% in The Cancer Genome Atlas (TCGA) PTC cohort (9). Analyses of molecular features from PTC tumors in TCGA have revealed that BRAF^mut^ tumors tend to express a lower abundance of markers of thyrocyte differentiation compared to BRAF^wt^ PTC (12). We previously published a transcriptomic analysis of a small cohort of patients with BRAF tumors (12 BRAF^mut^, 8 BRAF^wt^) and from these data posited that BRAF^mut^ tumors are immune-suppressive (23). Conversely, a larger TCGA study has subsequently revealed that BRAF^mut^ tumors express higher levels of several immune cell marker transcripts, consistent with an immune-activated state (24). However, a proteomic analysis of 30 PTC tumors (15 each BRAF^mut^ and BRAF^wt^) led to the conclusion that BRAF^mut^ tumors express lower levels of major histocompatibility complex class II (MHCII) components, indicative of immune suppression (25). In that study, it was proposed that down-regulation of MHCII engendered escape from immune surveillance, which could be relieved by BRAF inhibitors in preclinical models. Thus, it appears that although there is a link between BRAF^mut^ and the immune system, it is unclear whether BRAF^mut^ tumors are more or less immunogenic than BRAF^wt^ tumors.

The analyses described herein were carried out to evaluate immune gene expression in an independent cohort of patients with PTC tumors from the Mayo Clinic Thyroid Cancer Registry. An immune function gene expression platform (NanoString Technologies, Inc) was used to analyze 770 immune transcripts as a function of BRAF^mut^pTERT^mut^ status in 147 formalin-fixed, paraffin-embedded (FFPE) PTC tumor samples. The results were validated using the PTC cohort in TCGA. The results of our study reveal a novel, heretofore unappreciated, link between TERT and the immune system, a link which may account for the more aggressive phenotype of BRAF^mut^pTERT^mut^ PTC.

## 2 Materials and methods

### 2.1 Study approval

Our study was conducted in accordance with recognized ethical guidelines, including the US Common Rule, and Mayo Clinic Institutional Review Board approval was obtained in accordance with a filed assurance approved by the Department of Health and Human Services.

### 2.2 Patients and sample preparation

We reviewed the Mayo Clinic thyroid cancer database and identified patients with PTC who were treated for this diagnosis at Mayo Clinic in Jacksonville, Florida (MCF) between January 1^st^ 2001 and December 31^st^ 2015. Medical records of these patients were reviewed for the following clinical characteristics: demographic characteristics, pathology, presence of local or distant persistent/recurrent disease, and clinical outcome. We retrieved 147 FFPE samples from the Mayo Clinic biorepository. The original histology was reviewed and the degree of lymphocyte infiltration into the tumor stroma (TIL) was determined by a single endocrine pathologist (MR). TIL was graded as 0 (no inflammation) 1 (10% or less), 2 (11-30%) or 3 (>30%) of tumor involved by chronic inflammation. BLT in uninvolved thyroid tissue was also scored and reported as absent (0), mild (1) (scattered/lymphoid aggregates), moderate (2) (easily identifiable lymphoid aggregates), or marked (3) (large confluent lymphoid aggregates involving most of the tissue).

### 2.3 Mutation status determination and immune-related gene expression profiling

Ten-μm-thick sections from formalin-fixed paraffin-embedded archival tissue blocks were cut and fixed to glass microscopy slides. For each block, an additional consecutive 5-μm-thick section was stained with hematoxylin and eosin (H&E) and used as a template for identification of tumor and stroma on the corresponding unstained slides. In the Mayo cohort, the BRAF status was determined by immunohistochemistry (IHC) as previously described (26). The IHC findings were then confirmed by digital droplet polymerase chain reaction (dd PCR) (QX200 Droplet Digital™ PCR BioRad). The presence of 10% or more of mutant alleles in a tumor was used as the cutoff for classifying it as *BRAF*mut; those with <10% mutant alleles were deemed *BRAF*wt. TERT promoter status was determined by ddPCR.

Based on the corresponding H&E slide, 10 µm FFPE sections from each tumor were macrodissected and used for simultaneous purification of DNA and RNA using QIAGEN All Prep kit (catalog no. 80 204). For all samples, total cellular RNA (at a minimum concentration of 10 ng/µl) was assessed for yield (NanoDrop 2000 Thermo Scientific). Immune-related gene expression profiling was performed utilizing the nCounter^®^ PanCancer Immune Profiling Panel comprised of 770 immune genes and associated controls (**Supplementary Table S1**). 200ng of each total RNA sample was prepared as per the manufacturer’s instructions. The nSolver analysis software version 4.0 was used for custom QC and normalization of the nCounter data.

For the TCGA cohort, patient characteristics and pre-processed gene expression data, which included the BRAF and TERT mutational status were downloaded from https://tcga-data.nci.nih.gov/tcga/tcgaDownload.jsp.

### 2.4 Differential gene expression analysis

Generalized linear model (GLM) was performed to access differential expression of individual genes in binary comparisons based on NanoString PTC data. The comparisons included 1) BRAF^mut^pTERT^wt^ *vs* BRAF^wt^pTERT^wt^; 2) BRAF^mut^pTERT^mut^ *vs* BRAF^wt^pTERT^wt^; 3) BRAF^mut^pTERT^mut^ *vs* BRAF^mut^pTERT^wt^; and 4) BRAF^wt^pTERT^mut^ *vs* BRAF^wt^pTERT^wt^. (**Supplementary Figure S1**). Log2 fold change (FC) (ratio of geometric mean) with 95% confidence interval (CI) was estimated by GLM. Benjamini & Yekutieli (BY) adjusted P-values were calculated. For effective validation, we selected all the genes from TCGA data (if their names showed in NanoString PTC data) and ran the same differential expression analysis (**Supplementary Figure S2**). *P* values < 0.05 were considered statistically significant. All analyses were performed using R software, version 3.6.2. TIL status was compared using 2-tailed Fisher’s exact test. Chi-square test of independence was used for comparison of categorical data and Kruskal-Wallis test to compare continuous variables across groups.

### 2.5 Pathway analysis

In order to gain further insight to what drives the suppression of the immune genes in the BRAF^mut^pTERT^mut^ samples compared to BRAF^mut^pTERT^wt^, we conducted pathway analyses of the two cohorts. The Gene Set Enrichment Analysis (GSEA) software is publicly available from the Broad Institute of MIT and Harvard University (http://software.broadinstitute.org/gsea/index.jsp). Gene counts from Mayo cohort and TCGA cohort were utilized for Hallmark, chemical and genetic perturbations (CGP), and Gene Ontology Biological Process (GOBP) analysis (version v2024.1). GSEA was performed using the gene permutation option and gene sets smaller than 15 or larger than 500 were excluded. The false discovery rate (FDR) with less than 0.25 (FDR < 0.25) was considered as significantly enriched. Additionally, GSEA was conducted using gene set signatures from MsigDB database, with enrichment scores (NES) calculated to elucidate biological pathways associated with BRAF^mut^pTERT^wt^ and BRAF^mut^pTERT^mut^ tumors.

### 2.6 Immune cell deconvolution analysis

The relative abundance of immune cell levels were estimated from the gene expression profiles using CIBERSORTx (CIBERSORTx (stanford.edu) We used the leukocyte signature matrix 22 (LM22) from CIBERSORTx to deconvolute and quantify the abundance of 22 immune cell types from the NanoString gene expression data. Quantile normalization was applied, and the data was run in absolute mode using 100 permutations. The analysis was repeated utilizing the TCGA PTC data. The resulting absolute abundance values for each immune cell type was compared between BRAF^mut^pTERT^wt^ and BRAF^mut^pTERT^mut^ using linear regression analysis. Immune cells with p-value <0.05 were reported statistically significant.

## 3 Results

### 3.1 Patient characteristics

A total of 147 patients treated for PTC at MCF were identified using our thyroid cancer registry. There were 107 females and 40 males with a median (range) age at diagnosis of 50.3 years (18.1-88.3) (**Supplementary Table S2**). Two patients with recurrent disease had their initial surgery elsewhere and were operated at MCF for locally recurrent disease. One patient had a thyroid lobectomy elsewhere demonstrating a 9.5 cm PTC and completion thyroidectomy at MCF 15 years later, with a 1.7 cm PTC found in the remaining lobe. The other 144 patients had their initial surgery at MCF. One hundred and eighteen (78%) pathology specimens were PTC classic variant, 23 (16%) encapsulated variant, 4 (3%) oncocytic variant, and 2 (1%) tall cell variant. The cohort of 147 samples included two specimens of cribriform morular thyroid carcinoma which was considered a histological subtype of PTC according to the 2017 WHO classification of tumors, and a distinct histological category per the 2022 WHO classification (**Supplementary Table S2**). Twenty patients did not have their subsequent follow-up at MCF after the initial diagnosis and treatment. The remaining 127 patients were followed over a median (range) of 163.5 months (2-325 months). For the 145 primary tumor samples, median (range) tumor size was 1.5 cm (0.6-6.5). Primary tumor size was unknown for one patient who was operated at MCF for recurrent disease. One hundred and twenty two patients were TNM stage I, 17 were stage II, five were stage III, and three were stage IVB. Fourteen patients died during follow-up, all from causes other than thyroid cancer. At the last follow-up, 109 patients did not have any evidence of disease, six had persistent, anatomically detectable local disease, and one had detectable distant disease.

### 3.2 BRAF and TERT mutational status and lymphocytic infiltrate profiles

**Supplementary Table S3** displays the BRAF and TERT mutational status of the samples comprised in the Mayo cohort. BRAF^mut^ was identified in 93 samples (63.3%), and 54 (36.7%) were BRAF^wt^. Ten of the BRAF^mut^ samples also harbored pTERT^mut^ (6 C228T and 4 C250T). The remaining 83 samples were BRAF^mut^pTERT^wt^. In 49 samples, neither BRAF^mut^ nor pTERT^mut^ were identified. pTERT^mut^ was detected in five BRAF^wt^ samples (2 C228T and 3 C250T).

TIL and BLT status are presented in **Supplementary Tables S2–S4**. BRAF^mut^pTERT^wt^ tumors had TIL scores significantly elevated: (>0, Fisher exact two-tailed *P*=.001) and (>1, *P*=.042) compared to BRAF^wt^pTERT^wt^. In contrast none of the pTERT^mut^ tumors had a TIL score of ≥ 2, irrespective of the BRAF status. There was no statistically significant difference in the BLT profile across groups (p=0.63) (**Supplementary Table S2**).

### 3.3 Differential gene expression

We initially evaluated differential expression of immune function genes between BRAF^mut^pTERT^wt^ and BRAF^wt^pTERT^wt^ tumors. Since this was our largest set of samples, we used BY.adj.*P* to identify immune function transcripts that were differentially expressed in BRAF^mut^pTERT^wt^ with a high degree of confidence. We identified 345 transcripts that were differentially expressed at *P*<0.05. After adjustment, 148 transcripts remained significantly differentially expressed Benjamini-Yekutieli adjusted *P* values (BY.adj.*P ≤* 0.05) (**Supplementary Table S5**). We found that most of these transcripts were more abundant in BRAF^mut^pTERT^wt^ tumors (127 induced *vs* 21 repressed) than in BRAF^wt^pTERT^wt^ tumor **Supplementary Figure S3** lists the 127 genes induced in the BRAF^mut^ tumors, which include the T-cell marker, CD4; myeloid marker, ITGAX/CD11c; macrophage marker, CHIT1; check point transcripts CD274/B7-H3 which were robustly overexpressed (BY.adj.*P*<0.05). In general, data indicates that BRAF^mut^pTERT^wt^ tumors are more immunogenic than BRAF^wt^pTERT^wt^ tumors as also demonstrated in **Supplementary Figure S1A**.

#### 3.3.1 BRAF^mut^-induced immune gene expression is generally reversed by pTERT^mut^

**Figure 1:**
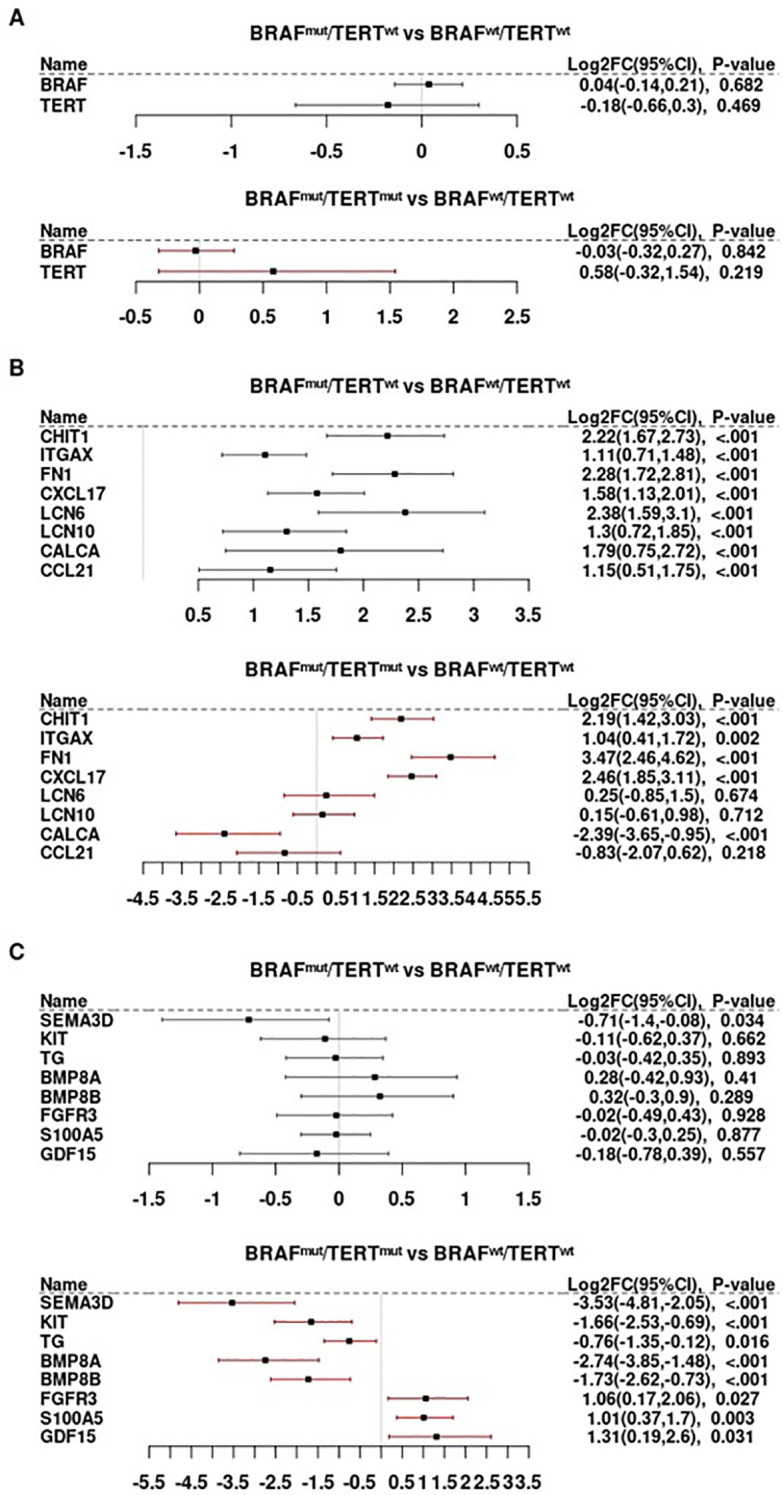
Differential gene expression as a function of BRAF and TERT mutational status. **(A)** BRAF and TERT mRNA abundance in relationship to the presence of BRAFV600E and TERT promoter mutations. **(B)** The TERT promoter mutations are associated with repression of CALCA, CCL21, strong induction of FN1, CXCL17, LCN6 and LCN10, and seems to have no effect on CHIT1 and ITGAX expression in BRAFV600E mutated tumors. **(C)** Immune genes whose expression is repressed in the presence of TERT promoter mutation but appears unaltered by the presence of BRAFV600E.

While BRAF^mut^ did not significantly affect the abundance of BRAF mRNA, pTERT^mut^, TERT mRNA abundance was significantly higher in BRAF^mut^pTERT^mut^ cells than in BRAF^mut^pTERT^wt^ (**Figure 1A**). Among the 127 immune transcripts induced by BRAF^mut^ in pTERT^wt^ tumors (log2 FC BRAF^mut^pTERT^wt^ *vs* BRAF^wt^pTERT^wt^ > 0, BY.adj.*P*<0.05), 87 transcripts were expressed at approximately the same level with pTERT^mut^ (BRAF^mut^pTERT^mut^ *vs* BRAF^mut^pTERT^wt^, *P*>0.05), while the other 40 transcripts were differentially expressed in BRAF^mut^pTERT^mut^ *vs* BRAF^mut^pTERT^wt^, either higher or lower (critical *P*<0.05) (**Supplementary Table S6**).

Most of the transcripts that were strongly induced by BRAF^mut^ in the absence of pTERT^mut^ were repressed in double mutant tumors (BRAF^mut^pTERT^wt^ > BRAF^mut^pTERT^mut^), as summarized in **Supplementary Figure S3**. However, the effect of BRAF^mut^ on some genes was unaltered by the addition of pTERT^mut^, as illustrated by the macrophage marker, CHIT1, and myeloid marker, IGTAX/CD11c (**Figure 1B**). The well-known BRAF^mut^ target, FN1/fibronectin, and monocyte/dendritic cell chemokine, CXCL17, were more potently induced in the double mutant tumors; however, some BRAF^mut^-induced genes (LCN6, LCN10) were not induced in BRAF^mut^pTERT^mut^ tumors and others (eg, CALCA/calcitonin and T-cell chemokine, CCL21) were suppressed in presence of pTERT^mut^ to levels below those observed in BRAF^wt^pTERT^wt^ tumors (**Figure 1B**). Moreover, several genes not significantly regulated by BRAF^mut^ were either repressed or induced in double mutant tumors as a function of TERT (**Figure 1C**).

**Figure 2:**
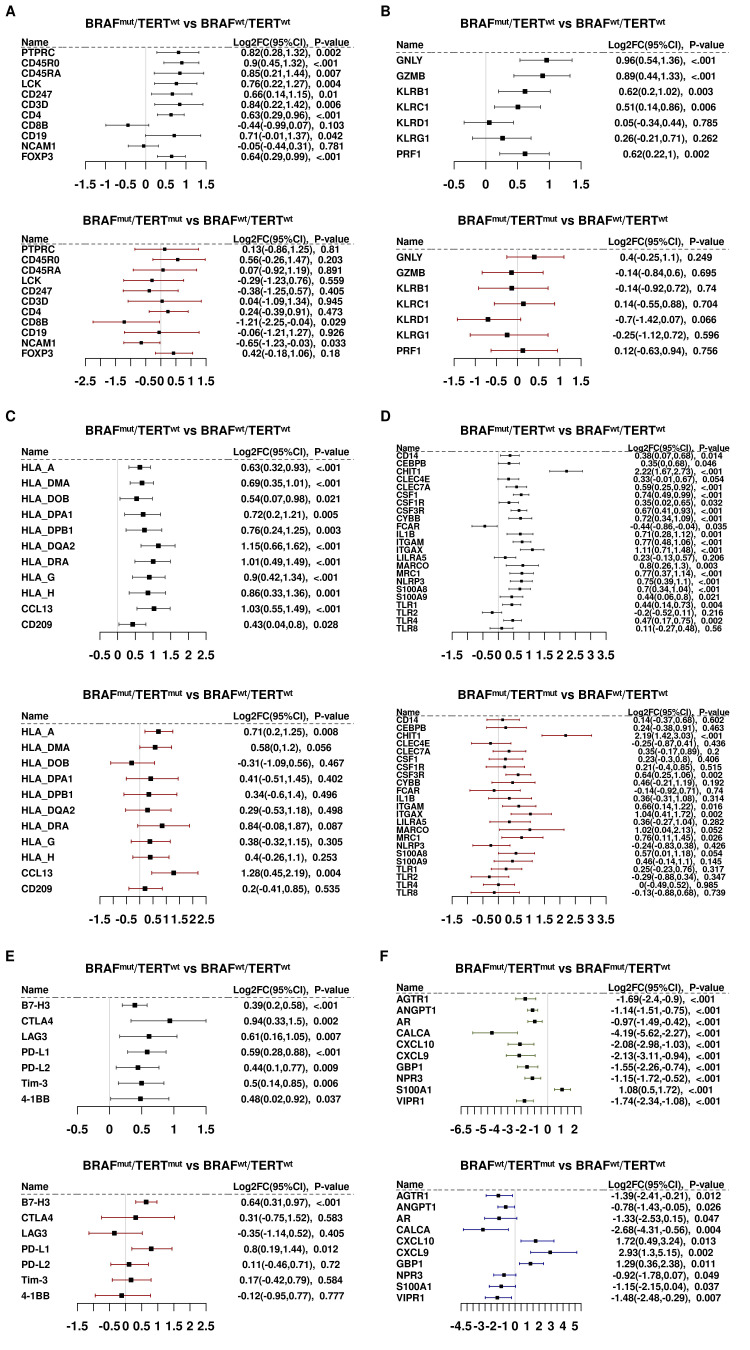
Panels **(A-E)**. Differential gene expression of immune gene subsets. **(A)** Lymphoid markers. **(B)** Cytotoxic cell markers. **(C)** Antigen presentation cell markers. **(D)** Myeloid markers. **(E)** Checkpoint Control I/IO drug targets. Panel **(F)**. Differentially expressed transcripts in 5 BRAF^wt^ samples harboring the TERT promoter mutation vs BRAF^wt^pTERT^wt^.

Lymphoid cell markers were generally induced in BRAF^mut^pTERT^wt^ but repressed in BRAF^mut^pTERT^mut^ tumors (**Figure 2A**). Notably the pan-leukocyte marker, PTPRC/CD45, and CD45 effector kinase, LCK, were less abundant in BRAF^mut^pTERT^mut^ tumors. The T-cell markers, CD247/CD3Z, CD4, and CD8B; B-cell marker, CD19; and putative natural killer (NK) cell marker, NCAM1/CD56, were repressed by pTERT^mut^. The Treg marker, FOXP3, was largely unaltered by pTERT^mut^. Consistent with the decreased CD8B and NCAM1/CD56 markers, we also noted decreased cytotoxicity markers in BRAF^mut^pTERT^mut^ tumors, including GZMB and NK cell markers, KLRB1, KLRC1 and KLRD1 (**Figure 2B**).

Genes associated with antigen presenting cells and myeloid cells did not appear to be significantly repressed by pTERT with the notable exceptions of the macrophage-activating marker, TLR4; monocyte activator, CSF1; and inflammasome subunit, NLRP3 (**Figures 2C, D**).

**Figure 2E** summarizes the effects of pTERT^mut^ on immune checkpoint genes and immuno-oncology targets. Expression of transcripts that encode two of the more actively studied checkpoint proteins, CD274/PD-L1 and CD276/B7-H3, were induced in BRAF^mut^ tumors and were not significantly affected by the addition of pTERT^mut^. Similarly, CTLA4 was induced by BRAF and was not significantly repressed by pTERT^mut^. Conversely, LAG3, which encodes a cell surface molecule expressed on activated T cells, NK cells, B cells and plasmacytoid cells and has been recently reported to drive T cell exhaustion, was markedly repressed in double mutant tumors (27, 28).

Given the observation that pTERT^mut^ significantly alters the BRAF^mut^-induced immune landscape, the question arises: is this the additive effect of two oncogenic mutations, or does the interaction of the two mutant genes lead to a molecular phenotype that is different from that imposed by either mutation alone? To address this question, we interrogated the immune landscape of a very small cohort of BRAF^wt^pTERT^mut^ PTC tumors (n=5). To this end, we compared transcripts that were differentially expressed (BY.adj.*P*<0.05) in BRAF^mut^pTERT^mut^ *vs* BRAF^mut^pTERT^wt^ with transcripts that were differentially expressed (critical *P*<0.05) in BRAF^wt^pTERT^mut^ *vs* BRAF^wt^pTERT^wt^. Only 10 genes were identified (**Figure 2F**), but the results suggest that pTERT^mut^ may directly repress some genes that were differentially expressed in BRAF^mut^pTERT^mut^ *vs* BRAF^mut^pTERT^wt^ (eg, CALCA/calcitonin, which is strongly induced in BRAF^mut^pTERT^wt^ and strongly repressed in BRAF^mut^pTERT^mut^). On the other hand, some genes appeared to be expressed in the double mutant tumors in a manner opposed to the effects of pTERT^mut^ alone (eg, interferon target genes, CXCL9, CXCL10, and GBP1). Given the very small sample cohort available for analysis, any conclusions must be advanced with caution. Nevertheless, there is some suggestion that BRAF^mut^ and pTERT^mut^ may interact in a fashion that results in an immune landscape that is different from that imposed by either mutation acting alone.

#### 3.3.2 Signaling activity

**Figure 3:**
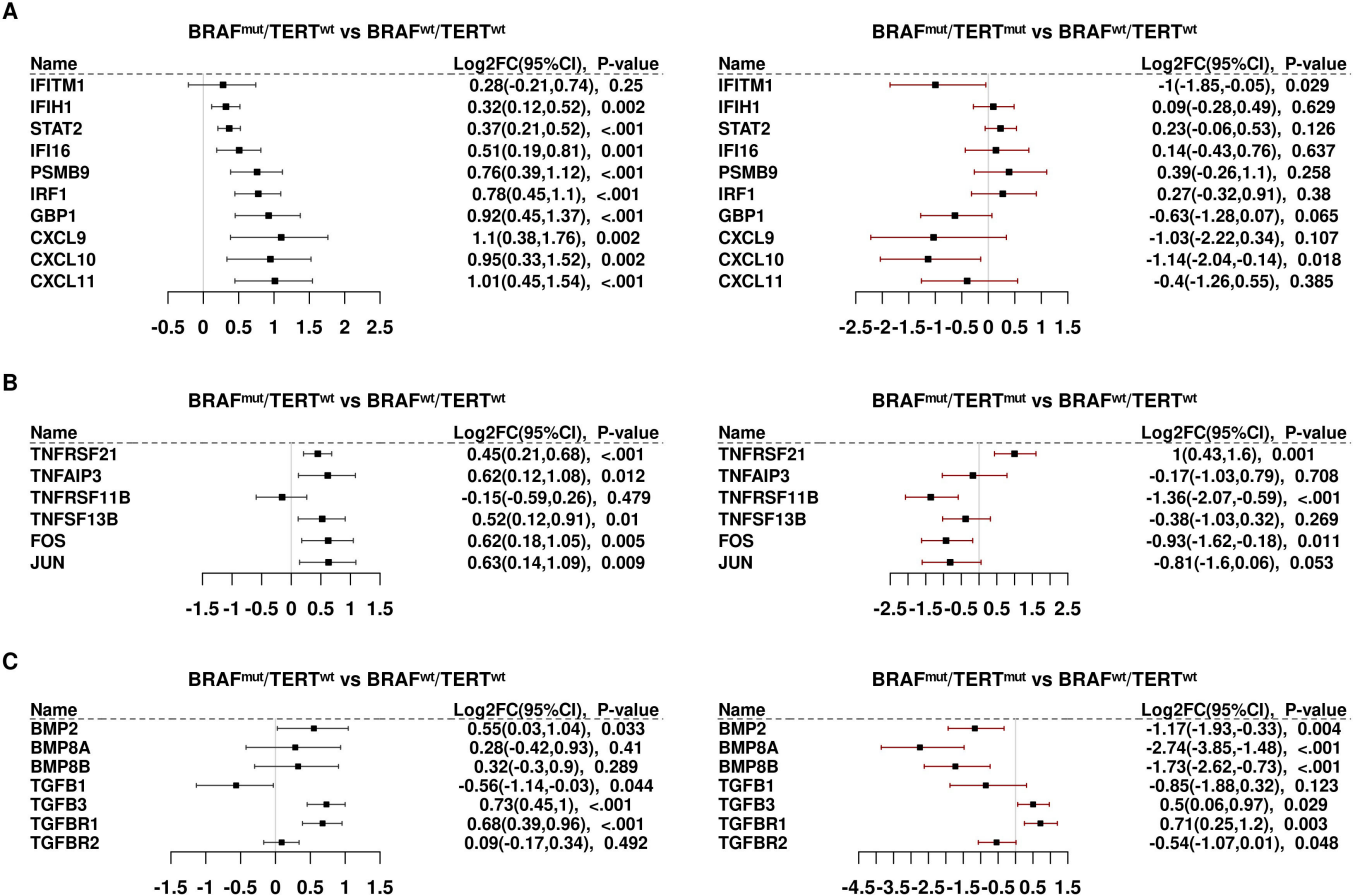
Differential expression of genes involved in signaling activity: **(A)** Interferon (IFN) downstream markers. **(B)** Tumor necrosis factor/TNF/AP1 signaling. **(C)** Bone morphogenetic proteins/transforming growth factor beta (BMP/TGFB).

Several interferon target genes were differentially expressed, generally induced in BRAF^mut^pTERT^wt^ and repressed in BRAF^mut^pTERT^mut^ samples (**Figure 3A**). Notable among these transcripts are CXCL9,10, and 11, which are ligands for the tumor-suppressive CXCR3 receptor. CXCR3 is also induced by BRAF^mut^ (**Supplementary Table S5**) but is not induced in double mutant tumors. A similar pattern prevailed with TNF superfamily transcripts, as well as AP1 subunits, FOS and JUN (**Figure 3B**). BMP markers were strongly repressed by pTERT^mut^; whereas TGFB1, TGFB2, and TGFBR1 were induced by BRAF^mut^ and unaffected by pTERT^mut^. However, the catalytic subunit of the TGFB receptor, TGFBR2, was significantly repressed by pTERT^mut^ (**Figure 3C**).

#### 3.3.3 Analysis of TCGA RNAseq data

Of the 496 patients with PTC in TCGA, the 444 with known BRAF and TERT status were included as our validation cohort (**Supplementary Tables S7, S8**). While BRAF^mut^ is relatively common in PTC, present is approximately half of all PTC tumors, pTERT^mut^ is rare. Thus, the number of BRAF^mut^pTERT^mut^ tumors available for analysis in our Mayo Clinic cohort was small, only 10 samples. We sought to validate our major findings using RNAseq data from our TCGA PTC cohort (**Supplementary Table S9**). Distribution of the various genotypes in TCGA samples is shown in **Supplementary Table S8**, noting that genotypes in this cohort were defined by sequence analysis of DNA extracted from fresh-frozen tumors; whereas, genotypes in the Mayo Clinic cohort were determined by dd PCR and IHC for BRAF^mut^ and dd PCR for pTERT^mut^ using DNA from FFPE samples. Transcript abundance in TCGA was determined by RNAseq analysis of fresh frozen RNA; while transcript abundance in Mayo Clinic samples was determined by NanoString analysis of RNA from FFPE samples. With these potentially confounding variables in mind, we sought to determine if the general pattern of immunosuppression observed in the double mutant samples from Mayo Clinic was recapitulated in the TCGA cohort. **Supplementary Figure S2** displays comparisons between the differential gene expression in BRAF^mut^pTERT^wt^ *vs* BRAF^w^tpTERT^wt^ (A), BRAF^mut^pTERT^mut^ *vs* BRAF^wt^pTERT^wt^ (B), BRAF^mut^pTERT^mut^ *vs* BRAF^mut^pTERT^wt^ (C) and BRAF^wt^pTERT^mut^ *vs* BRAF^wt^pTERT^wt^ (D) cohorts.

**Figure 4:**
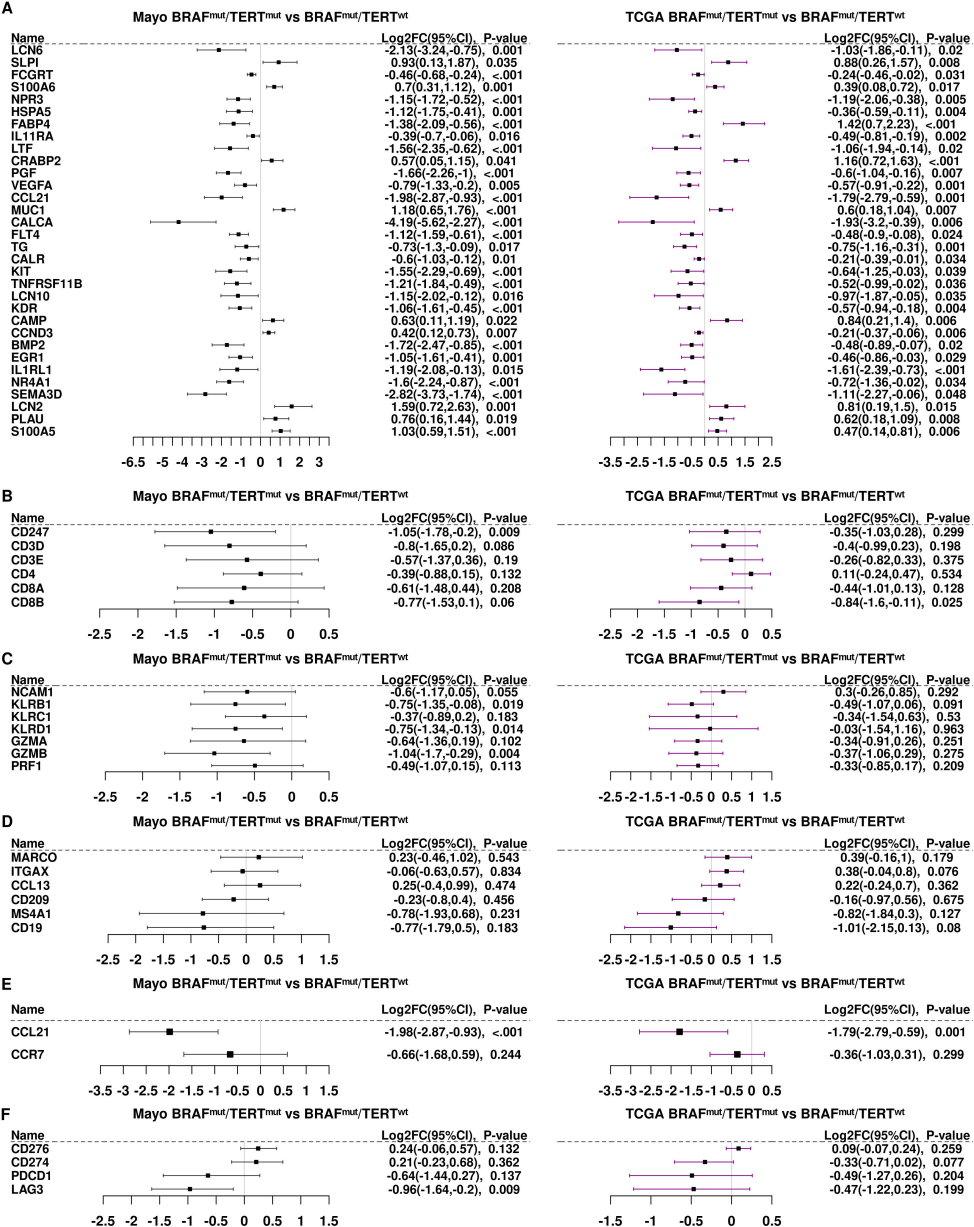
Differential gene expression of immune genes in the TCGA samples compared to the Mayo Clinic cohort. **(A)** We identified 40 immune genes significantly differentially expressed (p<0.05) in both the Mayo Clinic cohort (black bars) and in the TCGA samples (purple bars). Panels **(B-F)**. Differential gene expression of immune gene subsets: **(B)** T cell markers; **(C)** NK/cytotoxic cell markers; **(D)** antigen presenting cell markers; **(E)** CCL21 and CCR7 and **(F)** checkpoint genes.

**Figure 4A** shows the 40 transcripts that were strongly suppressed by pTERT^mut^ on the BRAF^mut^ background (log2 FC TCGA BRAF^mut^pTERT^mut^ *vs* BRAF^mut^pTERT^wt^< −0.5, *P*<0.05) in the Mayo Clinic samples being also suppressed in the TCGA cohort. As shown in **Figure 4A**, essentially all these transcripts were repressed in both TCGA and Mayo Clinic samples. Likewise, the T-cell receptor subunits, CD247/CD3Z, CD3D, and CD3E, were repressed by pTERT^mut^ in both datasets (**Figure 4B**). CD4 was slightly less abundant, albeit not significantly in Mayo Clinic patients, and CD4 was essentially unchanged in TCGA samples. Strikingly, markers of cytotoxic CD8+ T-cells (CD8A and CD8B) were strongly downregulated by pTERT^mut^ in both Mayo Clinic and TCGA samples (BRAF^mut^pTERT^mut^ *vs* BRAF^mut^pTERT^wt^) (**Figure 4B**). The NK cell markers, KLRB1 and KLRC1, as well as cytotoxic cell markers, GZMA, GZMB, and PRF1, exhibited similar patterns of regulation in the two sample cohorts (**Figure 4C**). A puzzling exception was NCAM1/CD56, which was downregulated in the Mayo Clinic pTERT^mut^ samples but not significantly altered in TCGA samples. It should be noted that, in addition to being an NK cell marker, CD56 is a marker of neuroendocrine differentiation, and therefore, not interpreted strictly as an immune cell marker in PTC tumors (29).

Similarities in the patterns of expression were observed regarding macrophage and myeloid cell markers, MARCO and ITGAX/CD11c, genes expressed by antigen presenting cells such as CCL13 and CD209. B-cell markers, MS4A1/CD20 and CD19, were strongly repressed in BRAF^mut^pTERT^mut^ tumors compared to BRAF^mut^pTERT^wt^ in both sample cohorts (**Figure 4D**).

CCL21, the second most highly repressed gene in TCGA BRAF^mut^pTERT^mut^ samples, was also strongly repressed in the Mayo Clinic cohort (**Figure 4E**). CCR7, the receptor for CCL21, was somewhat less abundant in BRAF^mut^pTERT^mut^ *vs* BRAF^mut^pTERT^wt^ in both sample cohorts. The CCL21/CCR7 axis is known to function in recruitment of cytotoxic cells (30) and downregulation of this pathway in double mutant cells may account, at least in part, for the observed decrease in cytotoxic markers in double mutant tumors. As shown in **Figure 4E**, the checkpoint factors, CD276/B7-H3 and CD274/PD-L1, were also equally abundant in both Mayo Clinic and TCGA samples. PCDC1/PD-1 and LAG3 were significantly repressed by pTERT^mut^ on the BRAF^mut^ background in both Mayo Clinic and TCGA samples (**Figure 4F**). Thus, the general patterns of regulation: pTERT^mut^ repression of CD8+ and NK cytotoxic cells with or dendritic cells; and relatively high levels of CD276/B7-H3 and CD274/PD-L1 were exhibited by both tumor sample cohorts.

### 3.4 Pathway analysis

**Figure 5:**
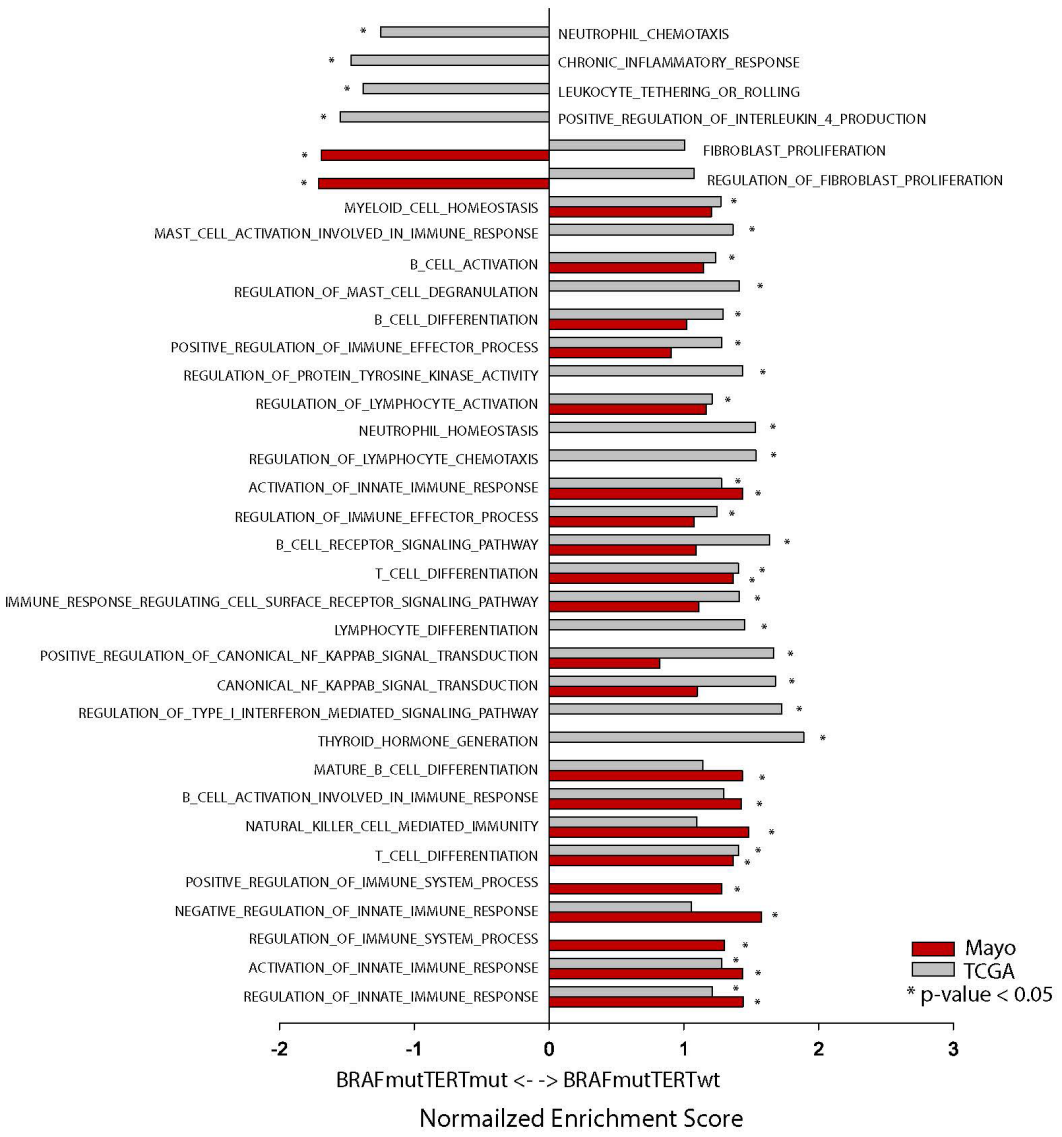
Pathway analysis of Mayo (red bars) and TCGA (gray bars) BRAF^mut^pTERT^wt^ *vs* BRAF^mut^pTERT^mut^ samples. * indicates p-value <0.05.

To evaluate the immune landscape function of the BRAF and TERT mutations, we performed pathway analysis of both Mayo and TCGA sample cohorts. (**Figure 5**, **Supplementary Table S10**). The overwhelming majority of immune related pathways were enriched in both BRAF^mut^pTERT^wt^ cohorts compared to the BRAF^mut^pTERT^mut^ cohorts. Four pathways were enriched in the BRAF^mut^pTERT^mut^ TCGA cohort: Positive regulation of IL-4 production, chronic inflammatory response, neutrophil chemotaxis and leukocyte tethering and rolling.

IL-4 is known to induce M2 macrophages which have immunosuppressive functions and have been linked to tumor progression (31–33). The TERT mutational status did not appear to influence the fibroblast proliferation and regulation of fibroblast proliferation pathways.

Despite the differences regarding sample preparation (fresh frozen *vs* FFPE), genotyping (DNA sequencing *vs* IHC and ddPCR), and transcriptome analysis (RNAseq *vs* NanoString), the data from both cohorts indicates that BRAF^mut^pTERT^mut^ tumors display a suppressed immune environment compared to BRAF^mut^pTERT^wt^.

### 3.5 Immune cell profiles

**Figure 6:**
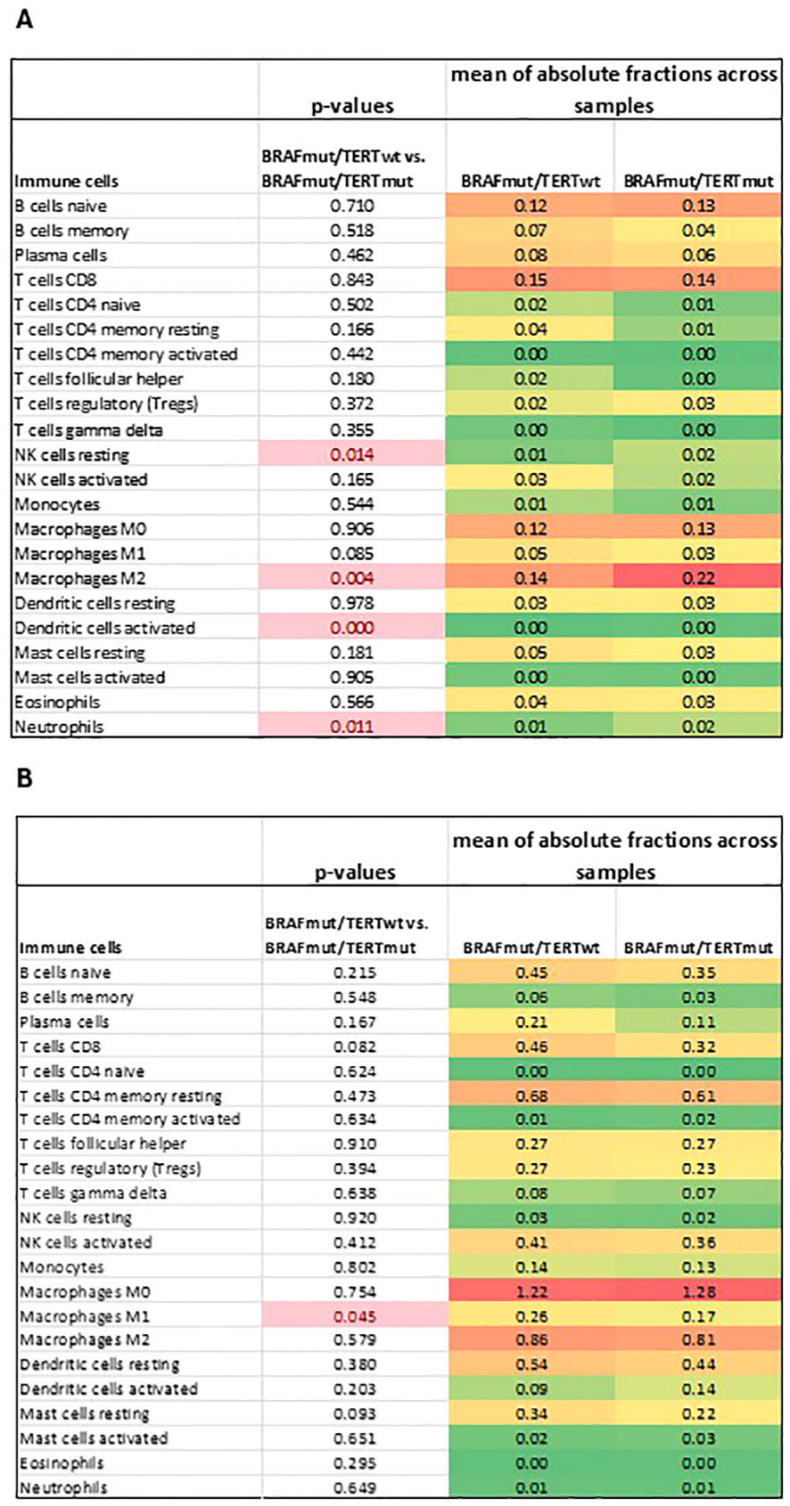
Deconvolution analysis of **(A)** Mayo and **(B)** TCGA BRAF^mut^pTERT^wt^ *vs* BRAF^mut^pTERT^mut^ sample cohorts. p-value <0.05 was considered statistically significant. ¥, mean of absolute fraction of activated dendritic cells in BRAF^mut^pTERT^wt^, 0.00006; &, mean of absolute fraction of activated dendritic cells in BRAF^mut^pTERT^mut^, 0.00137.

The differences between the immune cell composition between BRAF^mut^pTERT^wt^ and BRAF^mut^pTERT^mut^ was achieved by using CIBERSORTx, following the method proposed by Newman et al. (34), and summarized in **Figure 6**. In the Mayo cohort, the most notable observation was an increased proportion of the M2 macrophage in the BRAF^mut^pTERT^mut^ compared to the BRAF^mut^pTERT^wt^ (p<0.05). Additionally, the BRAF^mut^pTERT^mut^ demonstrated a reduced population of NK resting cells and increased neutrophils and activated dendritic cell populations. As this analysis was carried out with a limited set of 143 genes due to incomplete overlap of the two platforms (NanoString and CIBERSORTx) certain immune cell types may be underrepresented, therefore these results must be interpreted with caution. Absolute immune cell fractions in the individual pTERT^mut^ samples are listed in **Supplementary Figure S4** We repeated the same analysis utilizing the TCGA BRAF^mut^pTERT^wt^ and BRAF^mut^pTERT^mut^ sample set. While we did not observe a significant difference in the M2 macrophage population in the BRAF^mut^pTERT^mut^ cohort compared to the BRAF^mut^pTERT^wt^, the double mutant cohort exhibited a decreased proportion of the proinflammatory M1 macrophages. (**Figure 6B**) Absolute immune cell fractions in the individual pTERT^mut^ samples are presented in **Supplementary Figure S5**.

## 4 Discussion

To our knowledge, this is the first report of a potential link between TERT and the immune system in thyroid cancer. The *TERT* gene encodes the catalytic subunit of telomerase, which, in combination with the RNA subunit encoded by *TERC*, plays an important role in immortalization of cells. In thyroid cancer, *TERT* reactivation appears to be a late event in tumorigenesis, primarily the result of activating pTERT^mut^ and clinically associated with more aggressive disease (35). It is unclear whether the effects we observed are secondary to telomere elongation and immortalization, or whether there is some unknown function of TERT related to the immune system. In this context, it should be emphasized that the effects we observe in pTERT^mut^ tumors do not necessarily reflect direct actions of TERT but might be secondary consequences of telomere lengthening on the BRAF^mut^ background.

In an initial analysis of a small cohort of BRAF^mut^ PTC tumors (with no knowledge of pTERT status), we advanced the hypothesis that BRAF^mut^ suppressed the immune landscape of PTC tumors (23). A subsequent report from TCGA analysis, however, was inconsistent with this conclusion, reporting that myeloid cells, B-cells, and regulatory T-cells were significantly higher in the BRAF^mut^ group (12). The analysis reported herein is generally consistent with that TCGA publication. BRAF^mut^pTERT^wt^ PTC express significantly higher levels of immune markers linked to T-cells, B-cells, macrophage and dendritic cells. CD247 has been previously reported as downregulated in PTC tissue and postulated to attenuate NK cell–mediated toxicity (36). Downstream markers of interferon signaling were also markedly induced (GBP1, CXCL9, CXCL10, CXCL11), as were checkpoint control genes (CD276/B7-H3, CD274/PD-L1, CTLA4, and LAG3). Thus, the molecular data strongly suggest that BRAF^mut^pTERT^wt^ PTC tumors are associated with an immune-enriched microenvironment compared to BRAF^wt^ tumors. Further, analysis of stromal tumor-infiltrating lymphocytes is consistent with this conclusion.

BRAF^mut^ is common in PTC; most PTC sample cohorts contain about half BRAF^mut^ tumors. Conversely, pTERT^mut^ is rare in PTC; only 10 of 147 Mayo Clinic samples and 26 of 444 TCGA samples were BRAF^mut^pTERT^mut^. Thus, one must acknowledge from the outset that statistical power is limited for analysis of the double mutation genotype. This cautionary note applies even more to any attempt to analyze the exceedingly rare BRAF^wt^pTERT^mut^ PTC tumors. Nevertheless, the molecular landscapes of BRAF^mut^pTERT^wt^ and BRAF^mut^pTERT^mut^ tumors were strikingly different, allowing for interpretation of at least the more robust differences between these two genotypes.

pTERT^mut^ is believed to activate *TERT* transcription, and we observed a significant increase in TERT mRNA abundance in BRAF^mut^pTERT^mut^ cells. The abundance of BRAF mRNA did not appear to be affected by either BRAF or TERT mutation status. In general, pTERT^mut^ appeared to suppress the immune enriching effects of BRAF^mut^, although in some cases the gene expression did not seem influenced by TERT while in other cases the stimulatory effects of BRAF^mut^ were enhanced. Some genes were unaffected by BRAF^mut^ alone but were differentially expressed (compared to BRAF^wt^pTERT^wt^) in double mutant tumors. The effects of pTERT^mut^ on the BRAF^mut^ background were numerous and complex. To the extent that one may rely upon data from analysis of the extremely rare BRAF^wt^pTERT^mut^ tumors, it may be that some of the observed effects are simply additive; however, there is at least a suggestion that pTERT^mut^ and BRAF^mut^ may interact together to induce a molecular phenotype that is distinct from that imposed by either mutation alone. We noted that while *TERT* is strongly induced in BRAF^mut^pTERT^mut^ tumors, this gene is not differentially expressed in BRAF^wt^pTERT^mut^ tumors (compared to BRAF^wt^pTERT^wt^ tumors). Although the very small number of samples engenders caution, our data are consistent with a previous report that BRAF^mut^ induces *TERT* in pTERT^mut^ but not pTERT^wt^ tumors (37).

Despite the complexity of the observed difference in gene expression in BRAF^mut^pTERT^wt^ and BRAF^mut^pTERT^mut^ tumors, several important features clearly emerge. First, and perhaps the most important observation was the effect of pTERT^mut^ on the tumor associated macrophage (TAM) population. TAMs are generally categorized into classical activated macrophages, M1, and alternatively activated macrophages, M2, both able to be converted into each other in response to changes in the tumor microenvironment (38). While M1 macrophages have an anti-tumor effects, either by direct cytotoxic effect or by antibody-mediated cytotoxicity, M2 macrophages are immunosuppressive, promoting tumor progression, angiogenesis and invasion (32, 33, 38).

In our study, BRAF^mut^pTERT^mut^ tumors in the Mayo cohort displayed an increased proportion of M2 macrophages compared to the BRAF^mut^pTERT^wt^ group. This finding is supported by the down-regulation of TLR4. TLR4 deficiency has been associated with attenuation of adipose tissue inflammation, and promoting M2 macrophage polarization (39). In addition, the positive regulation of IL-4 pathway was enriched in BRAF^mut^pTERT^mut^ samples, IL-4 being a known activator of M2 macrophages (31, 40).

As the sample processing and methodology used to determine the genotype and transcript abundance Mayo and TCGA sample cohorts were different, our expectation was not to obtain the exact same results but to observe a similarly suppressed immune environment in the BRAF^mut^ pTERT^mut^ compared to the BRAF^mut^ pTERT^wt^. As such, while there was no significant difference in the M2 abundance in the TCGA cohort, the M1 macrophages were significantly less abundant in the BRAF^mut^pTERT^mut^ samples, indicative of a less immunogenic environment. A potential explanation may lay in the knowledge that activation of Pi3K signaling in TLR4 activated cells augments NF-κB activation, promoting M1 macrophage response (41). In our study, positive regulation of NF-κB pathway was suppressed in BRAF^mut^pTERT^mut^ compared to BRAF^mut^pTERT^wt^.

Second, profound effects of pTERT^mut^ were observed with B-cell markers (MS4A1/CD20 and particularly CD19) and markers of cytotoxic cells (CD8+ T-cells and NK cells). Key markers of these cell types are robustly and reproducibly (in Mayo Clinic and TCGA samples) repressed in double mutant tumors. It is plausible that these effects are linked to downregulation of interferon signaling, albeit effects on BMP and TNFSF signaling may also play a role (37). Third, the most striking effect of pTERT^mut^ on the BRAF^mut^ background was on the calcitonin gene, *CALCA*. This gene is strongly induced by BRAF^mut^ in Mayo Clinic samples and log2FC=-1.93 (critical p<0.006) in TCGA samples. *CALCA* encodes the neuropeptide αCGRP, which has been extensively studied as an anti-inflammatory signal (42). Thus, downregulation of *CALCA* should lead to inflammation, but this was not observed in BRAF^mut^pTERT^mut^ PTC tumors.

It is important to acknowledge that, despite seeing several markers being significantly downregulated, we did not observe corresponding major shifts in the immune cell populations. This could be explained by a decrease in the markers at individual level without driving a change in the cell populations, reflecting hyporesponsiveness of the immune effectors as seen with CD8 expression on cytotoxic T cells (43).

Our studies were motivated, at least in part, by the desire to identify features that account for the clinical observation that BRAF^mut^pTERT^mut^ PTC tumors are considerably more aggressive than BRAF^mut^pTERT^wt^ or BRAF^wt^pTERT^wt^ tumors (10). In a sense, our data appear to answer one question: why is the double mutant more aggressive than the BRAF^mut^pTERT^wt^ genotype? The answer plausibly lies within the immune microenvironment, which is clearly suppressed by pTERT^mut^. BRAF^mut^pTERT^wt^ tumors are more immunogenic that BRAF^mut^pTERT^mut^ tumors, and progression is more likely to be suppressed by the host immune system. Noncanonical functions of TERT include promoting epithelial to mesenchymal transition and cancer cell stemness (10, 44) as demonstrated by Caria et al. (45) in one BRAF^mut^pTERT^mut^ PTC cell line, B-CPAP. Recent studies suggest that the complex interplay between cancer stem cells and the immune microenvironment results in immunosuppression, and additional work is necessary to demonstrate if our findings can be explained through such mechanisms (12, 46). Moreover, double mutant tumors maintain relatively high levels of two potential immune-therapeutic targets, B7-H3 and PD-L1, which may inform novel therapeutic approaches for management of these more aggressive tumors. This is consistent with existing literature which reports that PDL1 expression is elevated in CD44+ breast and head and neck squamous cancer stem cells (37, 47, 48) and CD133+ colon cancer stem cells (49).

On the other hand, these data yield little insight into why double mutant PTC tumors are more aggressive than BRAF^wt^pTERT^wt^ tumors. Very few immune markers were repressed in BRAF^mut^pTERT^mut^ samples compared to BRAF^wt^pTERT^wt^ samples, so there does not seem to be a compelling immune explanation for clinical differences in aggressiveness between these two genotypes. This observation lends itself to speculation that we may be asking the wrong question. The key question may be: why is BRAF^mut^ not more oncogenic (ie, why are BRAF^mut^ PTC tumors not more aggressive than BRAF^wt^ tumors)? BRAF^mut^ constitutively activates growth factor/MAPK signaling, and from a signal transduction perspective, it is not obvious why BRAF^mut^ is not a powerful oncogene, in line with many well-known MAPK-activating oncogenic mutations to RAS, RAF, ERBB2, EGFR, etc. The answer to this enigma may lie in the observation that BRAF^mut^ engenders an environment that is immune-activated, thereby suppressing the transforming potential of the BRAFV600E oncogene. If this hypothesis is correct, one may understand how the pTERT mutation makes BRAF^mut^ tumors more aggressive, by suppressing the immune-activating properties of BRAF^mut^ while essentially unfettering the oncogenic driver stimulus that would normally be expected from a mutation that potentiates growth factor/MAPK signaling.

Although our data suggests that TERT is an important immune modulator in the tumor microenvironment, our methodology has two notable limitations. First, because both the NanoString Immune Profiling Panel and LM22 were comprised of a selected set of genes which did not overlap completely, the results of the deconvolution analysis of the Mayo samples may be under representative of certain immune cell types. However, the TCGA dataset deconvolution analysis was carried out with a 95% gene overlap. Second, our FFPE samples were macrodissected with the purpose of reducing the amount of normal tissue but did not remove it at a microscopic level. This prevented us from pursuing comparisons between immune gene expression in tumor versus background to further understand whether our findings are due to the result of gene alterations at the level of tumor cells, the surrounding immune cells or both. The answer to this question, as well as whether similar changes are present in other BRAF^mut^pTERT^mut^ thyroid cancer histologies will be the subject of future studies.

## Data Availability

The original contributions presented in the study are included in the article/**Supplementary Material**; further inquiries can be directed to the corresponding author/s.
