# Supplementary figures and images for: BRAFV600E/pTERT double mutated papillary thyroid cancers exhibit immune gene suppression
Source: Front Endocrinol (Lausanne). 2024 Dec 9;15:1440722. doi: 10.3389/fendo.2024.1440722 (PMC11663634; doi:10.3389/fendo.2024.1440722)

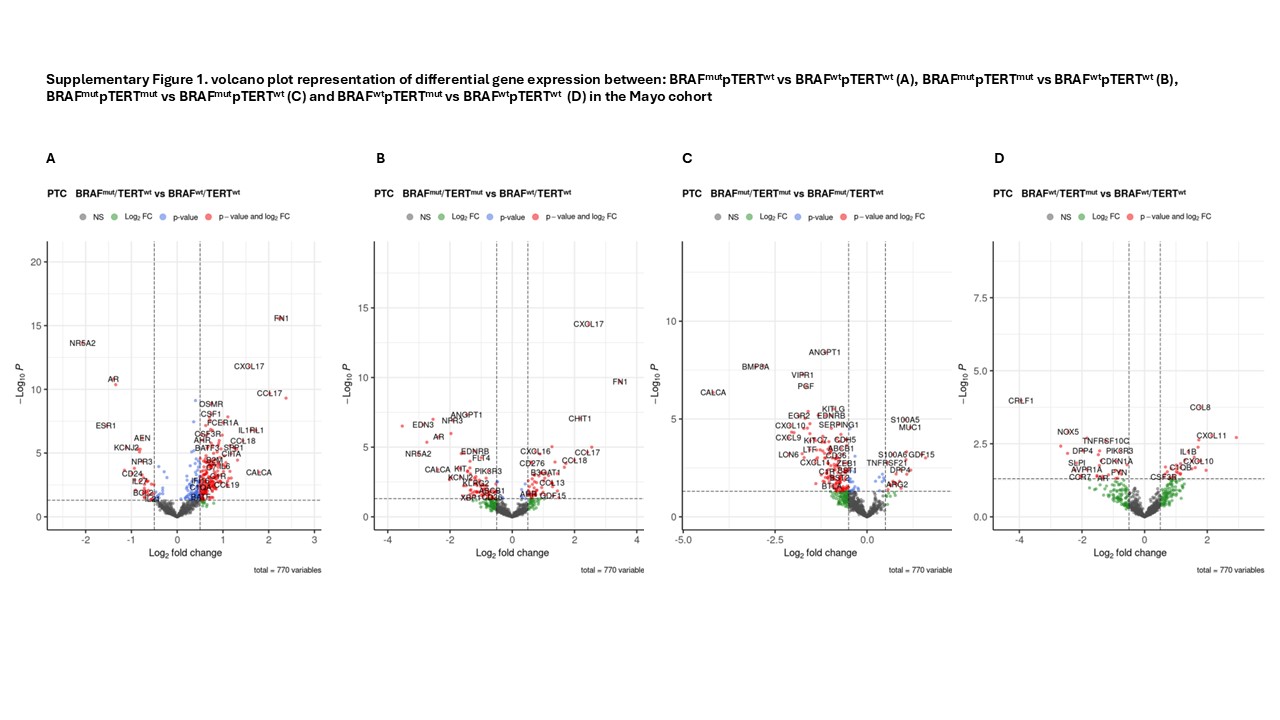

Supplement: Supplementary file 1 [file Image1.jpeg]

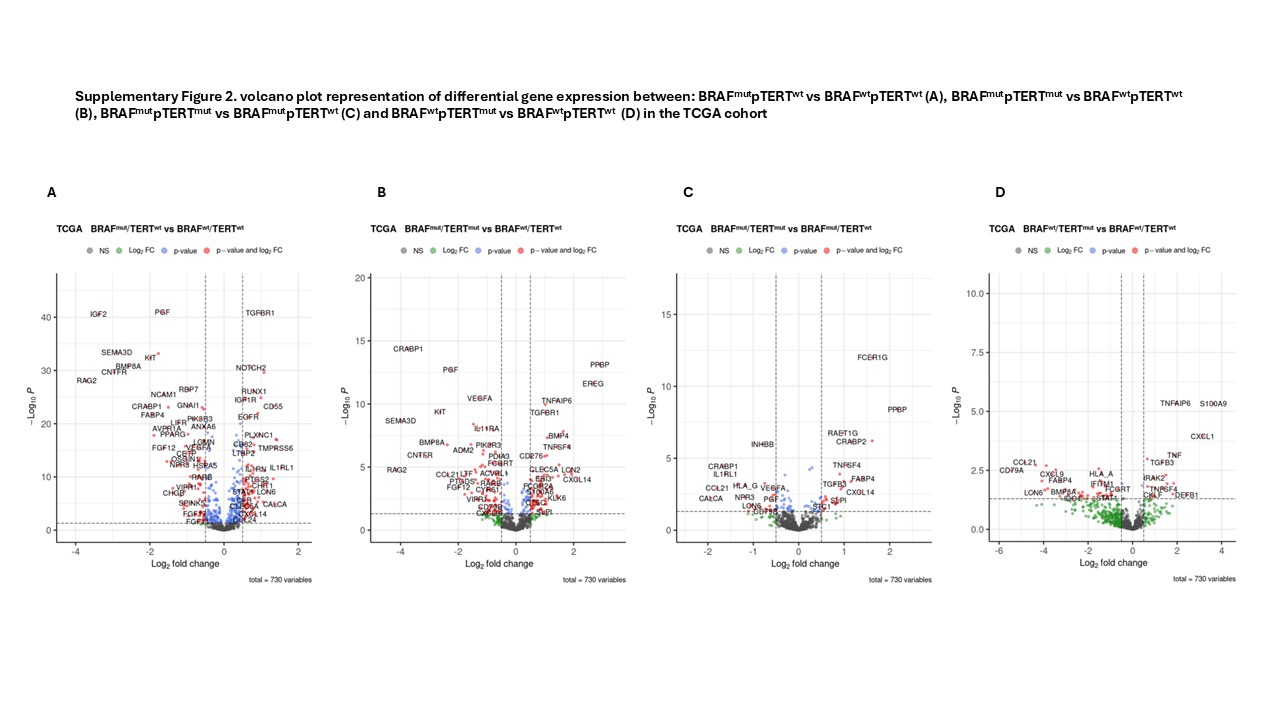

Supplement: Supplementary file 2 [file Image2.jpeg]

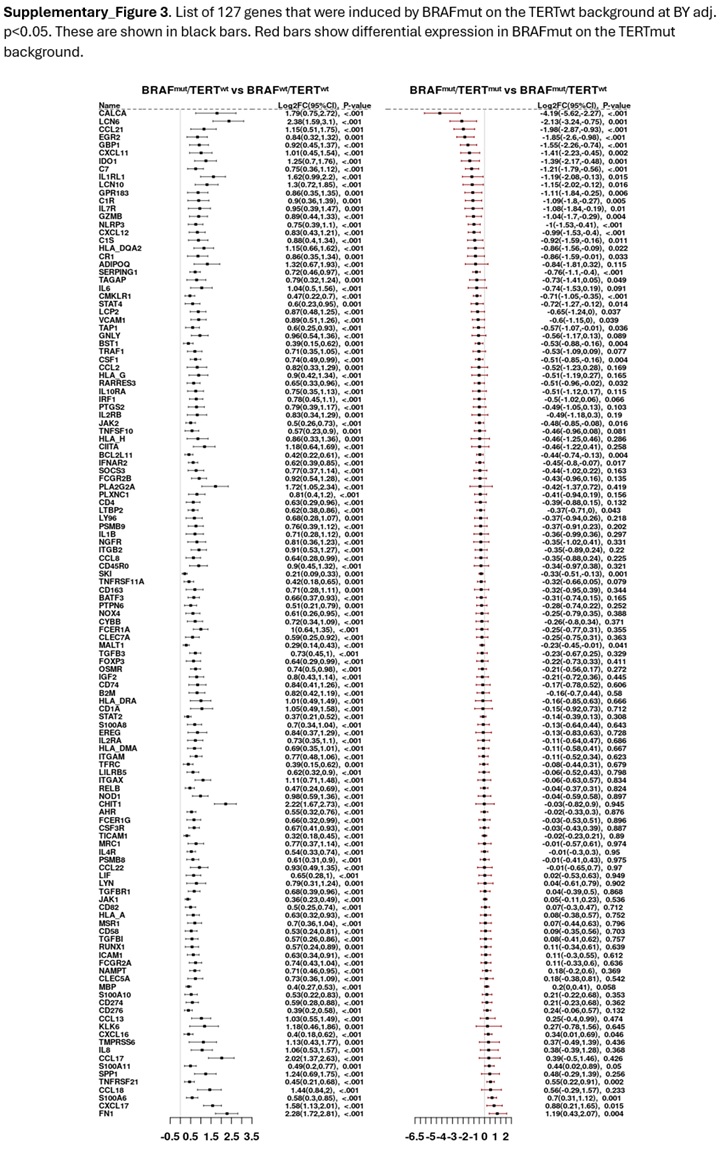

Supplement: Supplementary file 3 [file Image3.jpeg]

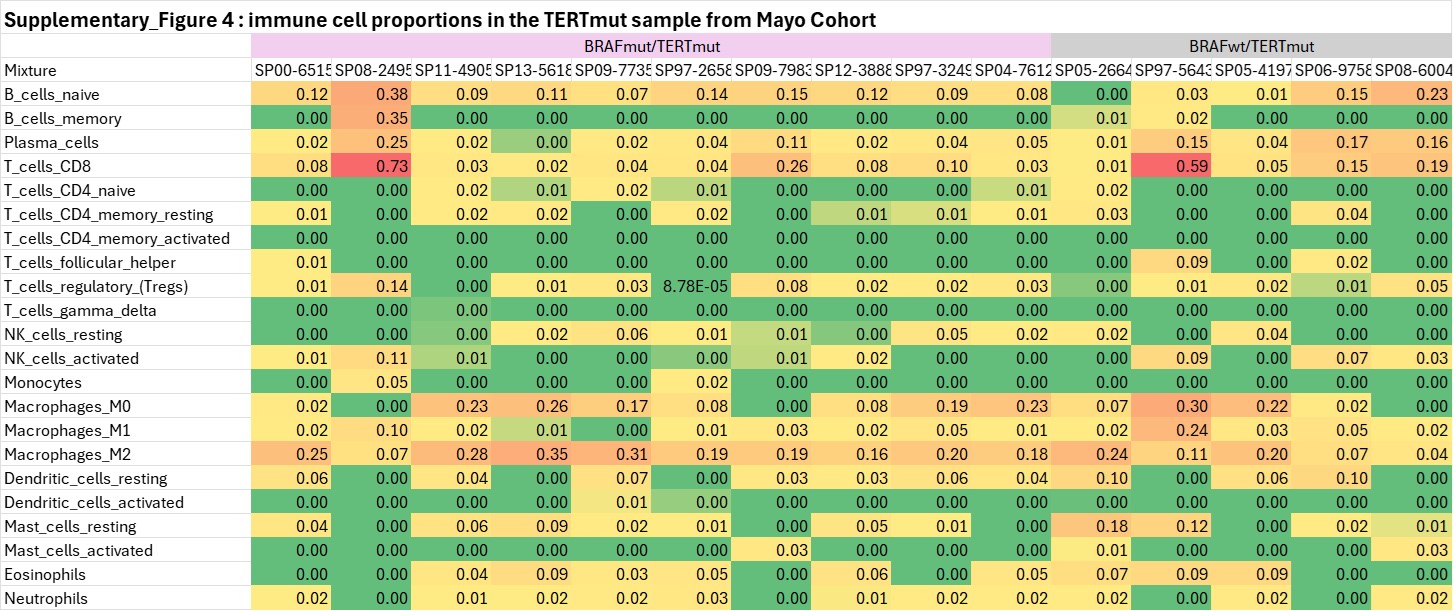

Supplement: Supplementary file 4 [file Image4.jpg]

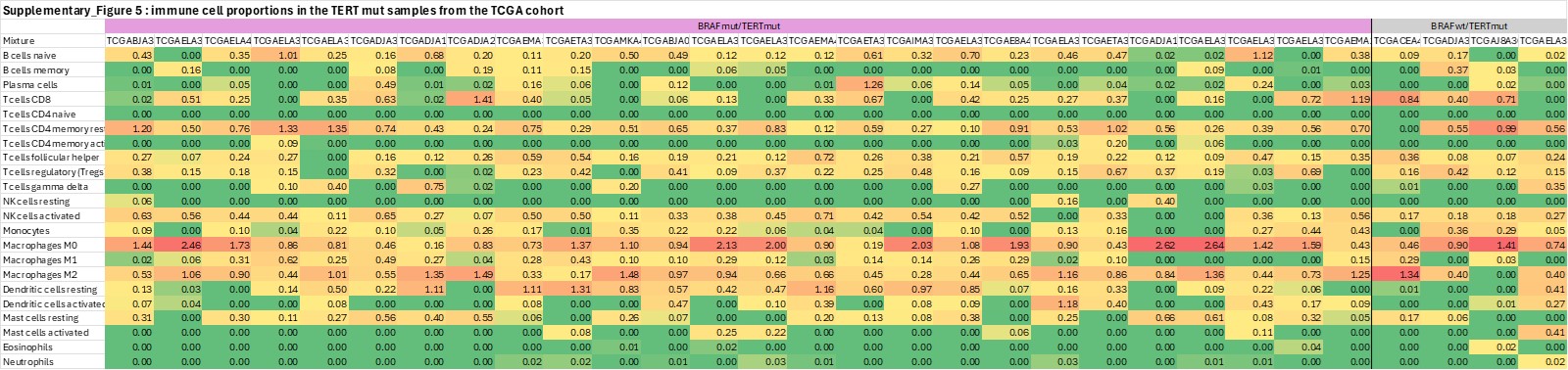

Supplement: Supplementary file 5 [file Image5.jpg]
