# Supplementary material for: BRAFV600E/pTERT double mutated papillary thyroid cancers exhibit immune gene suppression
Source: Front Endocrinol (Lausanne). 2024 Dec 9;15:1440722. doi: 10.3389/fendo.2024.1440722 (PMC11663634; doi:10.3389/fendo.2024.1440722)
Supplement: Supplementary file 7 [file Table2.docx]

**Supplementary Table 2: Demographic Description of Mayo Clinic Cohort**

|  | BRAFmutTERTwt (N=83) | BRAFmutTERTmut (N=10) | BRAFwtTERTwt (N=49) | BRAFwtTERTmut (N=5) | Total (N=147) | P_value |
| --- | --- | --- | --- | --- | --- | --- |
| **Age** |  |  |  |  |  | < 0.001 |
| Median (Range) | 48.65 (20.12, 84.56) | 66.21 (52.65, 81.22) | 46.77 (18.09, 83.27) | 61.50 (54.95, 88.28) | 50.31 (18.09, 88.28) |  |
| **gender** |  |  |  |  |  | 0.306 |
| F | 61 (73.5%) | 5 (50.0%) | 38 (77.6%) | 3 (60.0%) | 107 (72.8%) |  |
| M | 22 (26.5%) | 5 (50.0%) | 11 (22.4%) | 2 (40.0%) | 40 (27.2%) |  |
| **tumor.size** |  |  |  |  |  | 0.048 |
| Median (Range) | 1.50 (0.60, 6.50) | 2.80 (1.30, 6.50) | 2.00 (0.70, 6.50) | 1.50 (0.20, 1.80) | 1.50 (0.20, 6.50) |  |
| **T.stage** |  |  |  |  |  | < 0.001 |
| 1a | 21 (25.3%) | 0 (0.0%) | 13 (26.5%) | 0 (0.0%) | 34 (23.1%) |  |
| 1b | 28 (33.7%) | 3 (30.0%) | 15 (30.6%) | 0 (0.0%) | 46 (31.3%) |  |
| 2 | 11 (13.3%) | 2 (20.0%) | 17 (34.7%) | 0 (0.0%) | 30 (20.4%) |  |
| 3a | 2 (2.4%) | 0 (0.0%) | 2 (4.1%) | 1 (20.0%) | 5 (3.4%) |  |
| 3b | 19 (22.9%) | 3 (30.0%) | 0 (0.0%) | 2 (40.0%) | 24 (16.3%) |  |
| 4a | 2 (2.4%) | 2 (20.0%) | 2 (4.1%) | 1 (20.0%) | 7 (4.8%) |  |
| x | 0 (0.0%) | 0 (0.0%) | 0 (0.0%) | 1 (20.0%) | 1 (0.7%) |  |
| **N.stage** |  |  |  |  |  | < 0.001 |
| 0 | 26 (31.3%) | 2 (20.0%) | 9 (18.4%) | 4 (80.0%) | 41 (27.9%) |  |
| 0c | 21 (25.3%) | 2 (20.0%) | 20 (40.8%) | 0 (0.0%) | 43 (29.3%) |  |
| 1 | 1 (1.2%) | 1 (10.0%) | 1 (2.0%) | 0 (0.0%) | 3 (2.0%) |  |
| 1a | 25 (30.1%) | 0 (0.0%) | 14 (28.6%) | 0 (0.0%) | 39 (26.5%) |  |
| 1b | 10 (12.0%) | 4 (40.0%) | 5 (10.2%) | 0 (0.0%) | 19 (12.9%) |  |
| x | 0 (0.0%) | 1 (10.0%) | 0 (0.0%) | 1 (20.0%) | 2 (1.4%) |  |
| **M.stage** |  |  |  |  |  | < 0.001 |
| 0 | 83 (100.0%) | 9 (90.0%) | 47 (95.9%) | 2 (40.0%) | 141 (95.9%) |  |
| 1 | 0 (0.0%) | 1 (10.0%) | 2 (4.1%) | 3 (60.0%) | 6 (4.1%) |  |
| **TNM.stages** |  |  |  |  |  | < 0.001 |
| I+II | 82 (98.8%) | 8 (80.0%) | 47 (95.9%) | 2 (40.0%) | 139 (94.6%) |  |
| III+IV | 1 (1.2%) | 2 (20.0%) | 2 (4.1%) | 3 (60.0%) | 8 (5.4%) |  |
| **Follow.up months** |  |  |  |  |  | 0.318 |
| Median (Range) | 77.47 (0.00, 330.43) | 83.87 (0.03, 125.77) | 85.60 (0.23, 288.73) | 58.40 (4.23, 227.77) | 83.33 (0.00, 330.43) |  |
| **Background lymphocytic**  **thyroiditis** |  |  |  |  |  | 0.63 |
| N-Miss | 0 | 0 | 0 | 1* | 1* |  |
| 0 | 47 (57%) | 7 (70%) | 36 (74%) | 2 (50%) | 92 (63%) |  |
| 1 | 9 (11%) | 1 (10%) | 2 (4%) | 1 (25%) | 13 (9%) |  |
| 2 | 17 (20%) | 2 (20%) | 7 (14%) | 1 (25%) | 27 (18%) |  |
| 3 | 10 (12%) | 0 (0%) | 4 (8%) | 0 (0%) | 14 (10%) |  |
| **Histological Subtype** |  |  |  |  |  |  |
| Classic PTC | 74 (89%) | 9 (90%) | 31 (63%) | 1 (20%) | 115 (80%) |  |
| IFVPTC | 1 (1%) | - | - | - | 1 (1%) |  |
| O- PTC | 3 (4%) | - | 1 (2%) | - | 4 (3%) |  |
| Tall cell PTC | - | - | 2 (4%) | - | 2 (1%) |  |
| IEFVPTC | 5 (6%) | 1 (10%) | 13 (27%) | 4 (80%) | 23 (16%) |  |
| Cribriform | - | - | 2 (4%) | - | 2 (1%) |  |
| morular thyroid carcinoma |  |  |  |  |  |  |
| * no normal tissue was present in the sample | | | | | | |
