# Supplementary material for: BRAFV600E/pTERT double mutated papillary thyroid cancers exhibit immune gene suppression
Source: Front Endocrinol (Lausanne). 2024 Dec 9;15:1440722. doi: 10.3389/fendo.2024.1440722 (PMC11663634; doi:10.3389/fendo.2024.1440722)
Supplement: Supplementary file 9 [file Table4.docx]

**Supplementary Table 4 : Tumor Lymphocytic Infiltration (TIL) Scoring of 147 Mayo samples**

| **Genotype** | **TIL=0** | **TIL=1** | **TIL=2** | **TIL=3** |
| --- | --- | --- | --- | --- |
|  |  |  |  |  |
| BRAF^wt^/TERT^wt^  (N= 49) | 34 | 10 | 2 | 3 |
| BRAF^mut^/TERT^wt^  (N=83) | 33 | 29 | 12 | 9 |
| BRAF^mut^/TERT^mut^  (N=10) | 4 | 6 | 0 | 0 |
| BRAF^wt^/TERT^mut^  (N=5) | 1 | 4 | 0 | 0 |
| BRAF^wt^/TERT^wt^ 0 vs >0 against BRAF^mut^/TERT^wt^ 0 vs >0, Fisher Exact 2-tailed, p=0.001  BRAF^wt^/TERT^wt^ 0-1 vs >1 against BRAF^mut^/TERT^wt^ 0-1 vs >1, p=0.042  BRAF^wt^/TERT^wt^ 0 vs >0 against BRAF^mut^/TERT^mut^ 0 vs >0, p=0.14  BRAF^wt^/TERT^wt^ 0-1 vs >1 against BRAF^mut^/TERT^mut^ 0-1 vs >1, p=0.58 | | | | |
| N, number of samples; wt, wild type; mut, mutant | | | | |
