# Supplementary material for: BRAFV600E/pTERT double mutated papillary thyroid cancers exhibit immune gene suppression
Source: Front Endocrinol (Lausanne). 2024 Dec 9;15:1440722. doi: 10.3389/fendo.2024.1440722 (PMC11663634; doi:10.3389/fendo.2024.1440722)
Supplement: Supplementary file 12 [file Table7.docx]

# Supplementary Table 7 : Demographic description of TCGA cohort

|  | BRAFmutTERTwt (N=208) | BRAFmutTERTmut (N=26) | BRAFwtTERTwt (N=206) | BRAFwtTERTmut (N=4) | Total (N=444) | P_value |
| --- | --- | --- | --- | --- | --- | --- |
| **Age** |  |  |  |  |  | < 0.001 |
| Median (Range) | 42.50 (0.00, 78.00) | 65.00 (0.00, 89.00) | 44.50 (0.00, 88.00) | 52.50 (33.00, 69.00) | 45.00 (0.00, 89.00) |  |
| **gender** |  |  |  |  |  | 0.695 |
| 0 | 10 (4.8%) | 3 (11.5%) | 13 (6.3%) | 0 (0.0%) | 26 (5.9%) |  |
| FEMALE | 145 (69.7%) | 17 (65.4%) | 140 (68.0%) | 4 (100.0%) | 306 (68.9%) |  |
| MALE | 53 (25.5%) | 6 (23.1%) | 53 (25.7%) | 0 (0.0%) | 112 (25.2%) |  |
| **T.stage** |  |  |  |  |  | < 0.001 |
| 0 | 10 (4.8%) | 3 (11.5%) | 13 (6.3%) | 0 (0.0%) | 26 (5.9%) |  |
| T1 | 14 (6.7%) | 2 (7.7%) | 19 (9.2%) | 0 (0.0%) | 35 (7.9%) |  |
| T1a | 9 (4.3%) | 0 (0.0%) | 9 (4.4%) | 0 (0.0%) | 18 (4.1%) |  |
| T1b | 36 (17.3%) | 1 (3.8%) | 27 (13.1%) | 0 (0.0%) | 64 (14.4%) |  |
| T2 | 57 (27.4%) | 6 (23.1%) | 71 (34.5%) | 2 (50.0%) | 136 (30.6%) |  |
| T3 | 77 (37.0%) | 8 (30.8%) | 60 (29.1%) | 1 (25.0%) | 146 (32.9%) |  |
| T4 | 1 (0.5%) | 3 (11.5%) | 2 (1.0%) | 1 (25.0%) | 7 (1.6%) |  |
| T4a | 3 (1.4%) | 3 (11.5%) | 4 (1.9%) | 0 (0.0%) | 10 (2.3%) |  |
| TX | 1 (0.5%) | 0 (0.0%) | 1 (0.5%) | 0 (0.0%) | 2 (0.5%) |  |
| **N.stage** |  |  |  |  |  | 0.427 |
| 0 | 10 (4.8%) | 3 (11.5%) | 13 (6.3%) | 0 (0.0%) | 26 (5.9%) |  |
| N0 | 81 (38.9%) | 9 (34.6%) | 88 (42.7%) | 1 (25.0%) | 179 (40.3%) |  |
| N1 | 21 (10.1%) | 4 (15.4%) | 22 (10.7%) | 2 (50.0%) | 49 (11.0%) |  |
| N1a | 49 (23.6%) | 5 (19.2%) | 32 (15.5%) | 0 (0.0%) | 86 (19.4%) |  |
| N1b | 28 (13.5%) | 4 (15.4%) | 29 (14.1%) | 1 (25.0%) | 62 (14.0%) |  |
| NX | 19 (9.1%) | 1 (3.8%) | 22 (10.7%) | 0 (0.0%) | 42 (9.5%) |  |
| **M.stage** |  |  |  |  |  | 0.702 |
| [Not Available] | 0 (0.0%) | 0 (0.0%) | 1 (0.5%) | 0 (0.0%) | 1 (0.2%) |  |
| 0 | 10 (4.8%) | 3 (11.5%) | 13 (6.3%) | 0 (0.0%) | 26 (5.9%) |  |
| M0 | 115 (55.3%) | 13 (50.0%) | 95 (46.1%) | 3 (75.0%) | 226 (50.9%) |  |
| M1 | 3 (1.4%) | 1 (3.8%) | 2 (1.0%) | 0 (0.0%) | 6 (1.4%) |  |
| MX | 80 (38.5%) | 9 (34.6%) | 95 (46.1%) | 1 (25.0%) | 185 (41.7%) |  |
| **TNM.stages** |  |  |  |  |  | < 0.001 |
| N-Miss | 11 | 3 | 14 | 0 | 28 |  |
| I+II | 134 (68.0%) | 6 (26.1%) | 137 (71.4%) | 3 (75.0%) | 280 (67.3%) |  |
| III+IV | 63 (32.0%) | 17 (73.9%) | 55 (28.6%) | 1 (25.0%) | 136 (32.7%) |  |
